# Mitochondrial dysfunction-associated OPA1 cleavage contributes to muscle degeneration: preventative effect of hydroxytyrosol acetate

**DOI:** 10.1038/cddis.2014.473

**Published:** 2014-11-13

**Authors:** X Wang, H Li, A Zheng, L Yang, J Liu, C Chen, Y Tang, X Zou, Y Li, J Long, J Liu, Y Zhang, Z Feng

**Affiliations:** 1 Center for Mitochondrial Biology and Medicine, The Key Laboratory of Biomedical Information Engineering of the Ministry of Education, School of Life Science and Technology and Frontier Institute of Science and Technology, Xi'an Jiaotong University, Xi'an, China; 2 Tianjin Key Laboratory of Exercise Physiology & Sports Medicine, Tianjin University of Sport, Tianjin 300381, China

## Abstract

Mitochondrial dysfunction contributes to the development of muscle disorders, including muscle wasting, muscle atrophy and degeneration. Despite the knowledge that oxidative stress closely interacts with mitochondrial dysfunction, the detailed mechanisms remain obscure. In this study, *tert*-butylhydroperoxide (*t*-BHP) was used to induce oxidative stress on differentiated C2C12 myotubes. *t*-BHP induced significant mitochondrial dysfunction in a time-dependent manner, accompanied by decreased myosin heavy chain (MyHC) expression at both the mRNA and protein levels. Consistently, endogenous reactive oxygen species (ROS) overproduction triggered by carbonyl cyanide 4-(trifluoromethoxy) phenylhydrazone (FCCP), a mitochondrial oxidative phosphorylation inhibitor, was accompanied by decreased membrane potential and decreased MyHC protein content. However, the free radical scavenger *N*-acetyl-L-cysteine (NAC) efficiently reduced the ROS level and restored MyHC content, suggesting a close association between ROS and MyHC expression. Meanwhile, we found that both *t*-BHP and FCCP promoted the cleavage of optic atrophy 1 (OPA1) from the long form into short form during the early stages. In addition, the ATPase family gene 3-like 2, a mitochondrial inner membrane protease, was also markedly increased. Moreover, OPA1 knockdown in myotubes was accompanied by decreased MyHC content, whereas NAC failed to prevent FCCP-induced MyHC decrease with OPA1 knockdown, suggesting that ROS might affect MyHC content by modulating OPA1 cleavage. In addition, hydroxytyrosol acetate (HT-AC), an important compound in virgin olive oil, could significantly prevent *t*-BHP-induced mitochondrial membrane potential and cell viability loss in myotubes. Specifically, HT-AC inhibited *t*-BHP-induced OPA1 cleavage and mitochondrial morphology changes, accompanied by improvement on mitochondrial oxygen consumption capacity, ATP productive potential and activities of mitochondrial complex I, II and V. Moreover, both *t*-BHP- and FCCP-induced MyHC decrease was sufficiently inhibited by HT-AC. Taken together, our data provide evidence indicating that mitochondrial dysfunction-associated OPA1 cleavage may contribute to muscle degeneration, and olive oil compounds could be effective nutrients for preventing the development of muscle disorders.

Skeletal muscle is the largest amino acid pool and endocrine organ in the body, and its dysfunction reduces exercise capacity and overall health.^1^ The balance of protein synthesis and degradation sustains regular turnover, but immobilization,^2^ age,^3^ cancer cachexia^4, 5^ and strenuous exercise^6^ disrupt the balance and contribute to skeletal muscle disorders. Mitochondria work as energy stations and signal transducers to preserve normal organism function, and increasing evidence shows that mitochondrial dysfunction contributes to skeletal muscle disorders.^7, 8, 9^ Our previous study showed that strenuous exercise-induced muscle fatigue was accompanied by increased mitochondrial fission,^6^ and an increase in the mitochondrial fission marker dynamin-related protein (DRP1) was found in the skeletal muscle after a high-fat diet was consumed, accompanied by mitochondrial dysfunction and triglyceride accumulation.^10^ These studies indicate that disruption of mitochondrial dynamics plays a vital role during mitochondrial dysfunction-associated muscle disorders.

Optic atrophy 1 (OPA1) is localized on the inner mitochondrial membrane, faces the intermembrane space^11^ and controls mitochondrial inner membrane fusion and remodeling.^12^ Studies have indicated the presence of eight transcript variants,^13^ and OPA1 is differentially proteolyzed into two long and three short isoforms.^14, 15^ Different isoforms attach differently to the mitochondrial inner membrane to accurately remodel it and keep the dynamic balance. Previous studies have found that a decreased mitochondrial adenosine 5-triphosphate (ATP) level, generated by either apoptosis induction, mitochondrial membrane potential loss or inhibition of ATP synthase, is the common and crucial stimulus that controls OPA1 processing.^16, 17^ It is known that the L-forms of OPA1 undergo further processing in the matrix to produce the S-forms and that knockdown of OPA1-induced mitochondrial fragmentation is rescued by expression of the L-forms instead of the S-form of OPA1.^15^ In addition, the preservation of a stable pool of L-OPA1 at the inner mitochondrial membrane is reported to be sufficient to delay mitophagy and maintain significant mitochondria content.^18^ Regarding OPA1 cleavage, a series of proteases localized to mitochondria including the ATPase family gene 3-like 2 (Afg3l2) and high temperature requirement protein A2 (HtrA2) are reported to participate in and modulate mitochondria morphology and functional activity.^19, 20, 21^ Although several studies have indicated that OPA1-modulated mitochondrial fusion is vital to maintaining mitochondrial and muscle function in various ways,^22, 23^ studies on the OPA1 cleavage regulation of oxidative stress and muscle cell disorders remain limited.

Olive oil is an integral ingredient of the ‘Mediterranean diet', and accumulating evidence suggests that it may have several benefits with respect to cancer,^24^ metabolic syndrome,^25^ heart function^26^ and muscle function.^27^ The abundant phenolic compounds with antioxidant properties are suggested to make major contributions. Hydroxytyrosol (HT) and hydroxytyrosol acetate (HT-AC) are well-known polyphenolic compounds found in olive oil, and HT-AC showed better antioxidant activity than HT.^28^ In our previous study, we found that HT could prevent strenuous exercise-induced muscle dysfunction,^6^ as well as reduce muscle lipid accumulation in mice fed a high-fat diet.^10^ Since then, no studies have reported the effects of HT-AC on muscle disorders. In the current study, we investigated the involvement of OPA1 cleavage and the protective effect of HT-AC on oxidative stress-induced mitochondrial dysfunction and muscle degeneration in C2C12 myoblast cells.

## Results

### Oxidative stress induces rapid mitochondrial dysfunction in muscle cells

**Figure 1:**
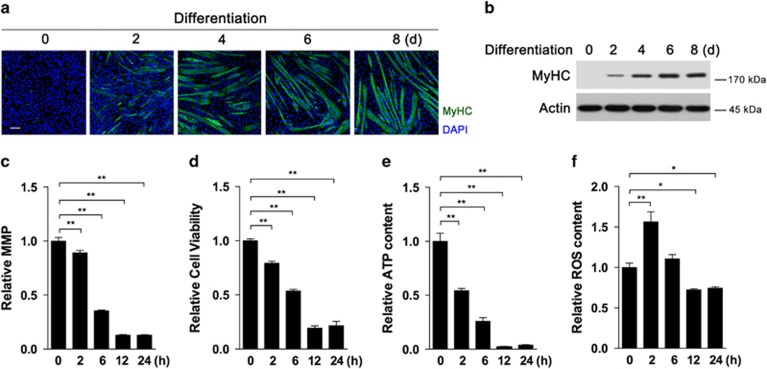
Oxidative stress induces rapid mitochondrial dysfunction in C2C12 muscle cells. C2C12 myoblasts were induced into myotubes after 8 days, and immunofluorescence (**a**) and western blot analysis (**b**) of MyHC were used to confirm the efficiency of differentiation. After 8 days of differentiation, myotubes were treated with 100 *μ*M *t*-BHP for the indicated time periods, and mitochondrial membrane potential (**c**), cell viability (**d**), cellular ATP content (**e**) and ROS levels (**f**) were detected. The values are means±S.E.M. from at least three independent experiments. *P<0.05, **P<0.01

To investigate the effects of oxidative stress on mitochondrial function in muscle cells, 100 *μ*M *tert*-butylhydroperoxide (*t*-BHP) was chosen to challenge differentiated C2C12 myotubes as an exogenous oxidative stress, following a previous work.^6^ The efficiency of myotube differentiation was verified by immunofluorescence and protein content analysis of myosin heavy chain (MyHC), a major marker of muscle cell, at different time points (Figures 1a and b). Myotubes were treated with *t*-BHP for the indicated time points (0, 2, 6, 12 and 24 h), and mitochondrial membrane potential (MMP), cell viability and cellular ATP content decreased in a time-dependent manner (Figures 1c–e). Similar decrease on cell viability and MMP was also observed in C2C12 myoblasts without differentiation (Supplementary Figure S1). After 2 h of treatment, *t*-BHP induced a marked increase in cellular reactive oxygen species (ROS) that generally declined afterwards (Figure 1e).

### Oxidative stress includes C2C12 myotubes degeneration

**Figure 2:**
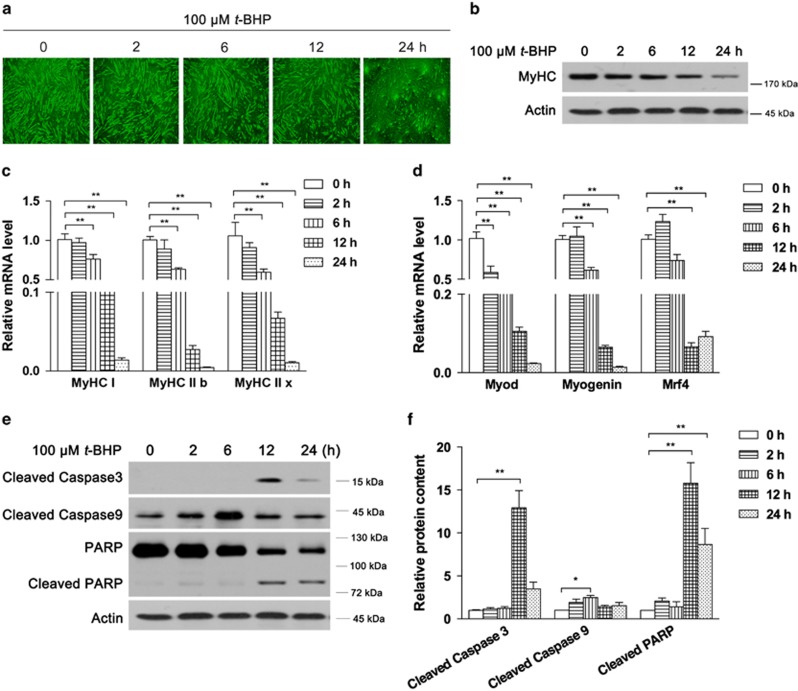
Oxidative stress induces muscle cell degeneration. After 8 days of C2C12 myoblast differentiation, myotubes were treated with 100 *μ*M *t*-BHP for the indicated time points. Cell morphology and myotube structure were observed by microscopy (**a**). MyHC protein was analyzed by western blotting (**b**). The mRNA contents of the MyHC isoforms MyHC I, MyHC IIb and MyHC IIx (**c**) and MyHC regulators Myod, Myogenin and Mrf4 (**d**) were analyzed by real-time PCR. Cell apoptosis activation was confirmed by analyzing cleaved caspase 3, cleaved caspase 9, PARP and cleaved PARP ((**e**) western blot image; (**f**) statistical analysis). The values are means±S.E.M. from at least three independent experiments. *P<0.05, **P<0.01

As shown in Figure 2a, t-BHP induced significant morphology changes in myotubes that became shorter and irregular after 24 h of treatment. Meanwhile, the protein content of MyHC was found to decrease in a time-dependent manner in C2C12 myotubes (Figure 2b). In addition, the mRNA expression levels of the MyHC isoforms, MyHC I, IIb and IIx, were also significantly decreased after 6 h of *t*-BHP treatment and were reduced by nearly 95% after 24 h (Figure 2c). As known transcriptional regulators of MyHC, mRNA levels of myogenic differentiation 1 (Myod), Myogenin and Mrf4 were also found decreased dose-dependently by *t*-BHP (Figure 2d). Similar decrease was also observed on Murf-1 and Atrogin-1, the key regulators of MyHC degradation and well-known markers of muscle atrophy (Supplementary Figure S2). Therefore, we proposed that *t*-BHP decreased MyHC content through transcriptional suppression. And, as expected, *t*-BHP induced cell apoptosis activation evidence by increased cleaved caspase 3, cleaved caspase 9 and cleaved PARP (Figures 2e and f).

### Mitochondrial ROS overproduction decreases MyHC expression

**Figure 3:**
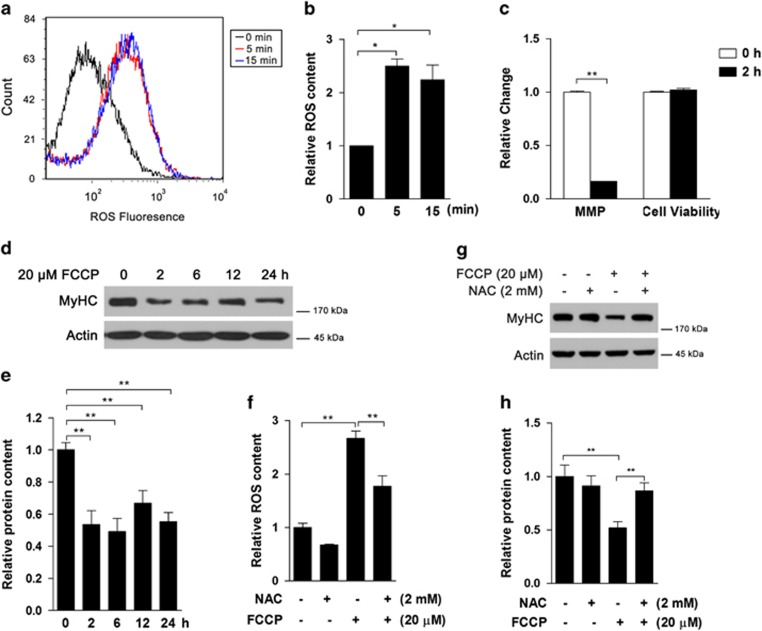
Mitochondrial dysfunction-induced ROS overproduction triggers MyHC decline. After 8 days of differentiation, C2C12 myotubes were treated with 20 *μ*M FCCP for 5 and 15 min, and the cellular ROS contents were analyzed by flow cytometry ((**a**) fluorescence curve; (**b**) statistical analysis). After 2 h of FCCP treatment, mitochondria membrane potential and cell viability was tested (**c**). At multiple time points after FCCP treatment, MyHC protein content was analyzed by western blot ((**d**) western blot image; (**e**) statistical analysis). Following pretreatment with 2 mM NAC for 30 min, C2C12 myotubes were treated with 20 *μ*M FCCP for 5 min to evaluate the ROS level (**f**), and for 2 h to test MyHC protein content by western blot ((**g**) western blot image; (**h**) statistical analysis). Values are means±S.E.M. from at least three independent experiments. *P<0.05, **P<0.01

To further investigate the relationship between mitochondrial dysfunction and myotube degeneration, carbonyl cyanide 4-(trifluoromethoxy) phenylhydrazone (FCCP), a mitochondrial uncoupler, was used to suppress mitochondrial function. FCCP at 20 *μ*M induced rapid ROS overproduction at 5 and 15 min (Figures 3a and b) and generally decreased after 2 h (data not shown). MMP was markedly decreased after FCCP treatment for 2 h, whereas cell viability was not affected (Figure 3c). Unlike *t*-BHP treatment, FCCP could sharply decrease MyHC protein content after 2 h (Figures 3d and e), and similar protein level was maintained from 6 to 24 h. *N-*acetyl-L-cysteine (NAC), the free radical scavenger, was used to eliminate excess ROS induced by FCCP (Figure 3f). As expected, the decrease in MyHC was sufficiently inhibited by NAC (Figures 3g and h).

### Activation of OPA1 cleavage is an early response to oxidative stress

**Figure 4:**
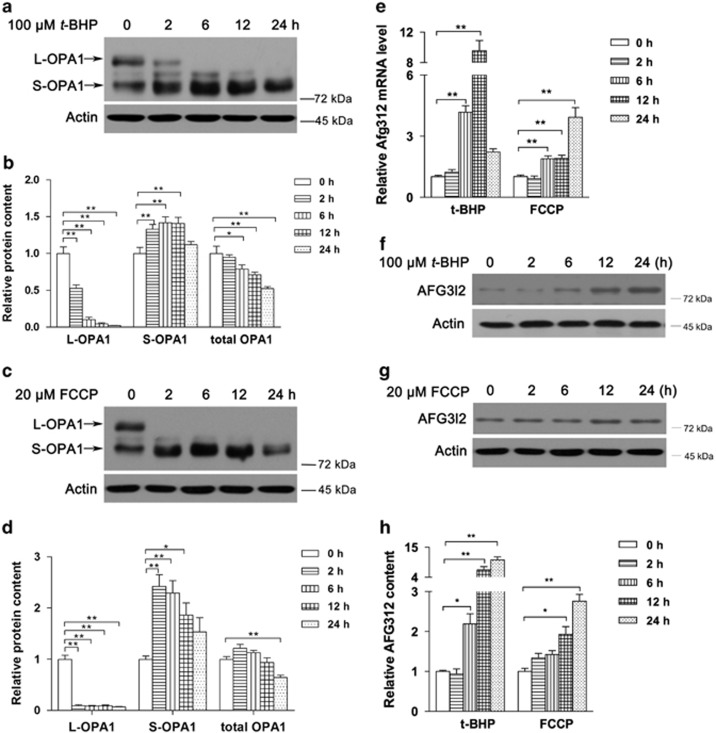
Activation of OPA1 cleavage is an early response of oxidative stress. C2C12 myotubes were treated with 100 *μ*M *t*-BHP or 20 *μ*M FCCP for the indicated time points, and then OPA1 levels and mitochondrial inner membrane proteases were analyzed. For *t*-BHP treatment, a western blot image of the L-OPA1 and S-OPA1 proteins (**a**) and associated statistical analysis (**b**). For FCCP treatment, a western blot image of the L-OPA1 and S-OPA1 proteins (**c**) and associated statistical analysis (**d**). (**e**) Afg3l2 mRNA level under both *t*-BHP and FCCP treatment. (**f**) A western blot image of AFG312 under *t*-BHP treatment, (**g**) a western blot image of AFG312 under FCCP treatment and associated statistical analysis (**h**). Values are means±S.E.M. from at least three independent experiments. *P<0.05, **P<0.01

Previous studies have indicated that decreased mitochondrial ATP or mitochondrial membrane potential loss is the common and crucial stimulus that controls OPA1 processing. Here, we found that *t*-BHP promoted rapid OPA1 cleavage from the long form of OPA1 (L-OPA1) into the short form of OPA1 (S-OPA1), accumulated after 2 h of treatment (Figures 4a and b). Consistently, FCCP treatment showed a similar pattern of OPA1 cleavage (Figures 4d and e). The mitochondrial inner protease Afg3l2, which participate in OPA1 cleavage, was found increased time-dependently after both *t*-BHP and FCCP treatment on mRNA levels (Figure 4e) and protein expression (Figures 4f–h).

### Oxidative stress decreases MyHC content through activation of OPA1 cleavage

**Figure 5:**
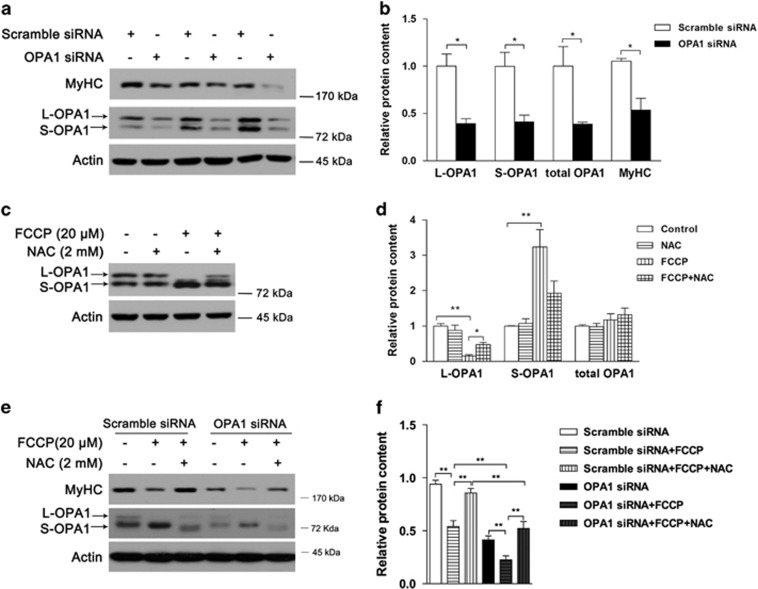
Oxidative stress decreases MyHC content through modulation of OPA1. C2C12 myotubes were transfected with OPA1 siRNA or with scrambled siRNA as negative control. After 72 h of transfection, OPA1 and MyHC protein contents were analyzed by western blotting ((**a**) western blot image; (**b**) statistical analysis). Following pretreatment with 2 mM NAC for 30 min, C2C12 myotubes were treated with 20 *μ*M FCCP for 2 h and OPA1 contents were analyzed ((**c**) western blot image; (**d**) statistical analysis). Myotubes were transfected with OPA1 siRNA for 72 h followed by 2 mM NAC or/and 20 *μ*M FCCP for another 2 h, and OPA1 and MyHC protein contents were analyzed ((**e**) western blot image; (**f**) statistical analysis). Values are means±S.E.M. from at least three independent experiments. *P<0.05, **P<0.01

To further investigate the effect of OPA1 on MyHC expression, specific OPA1 siRNA was used to knock down OPA1 expression, and MyHC protein level was found to be decreased significantly (Figures 5a and b). Because NAC was found to prevent FCCP-induced MyHC decline (Figure 3g), we were able to determine that NAC could significantly inhibit FCCP-induced OPA1 cleavage (Figures 5c and d). Meanwhile, the preventive effects of NAC on FCCP-induced MyHC decline were abolished by OPA1 siRNA (Figures 5e and f). The above data suggested that oxidative stress decreased MyHC content through activation of OPA1 cleavage.

### Protective effects of HT and HT-AC on *t*-BHP-induced cell toxicity

**Figure 6:**
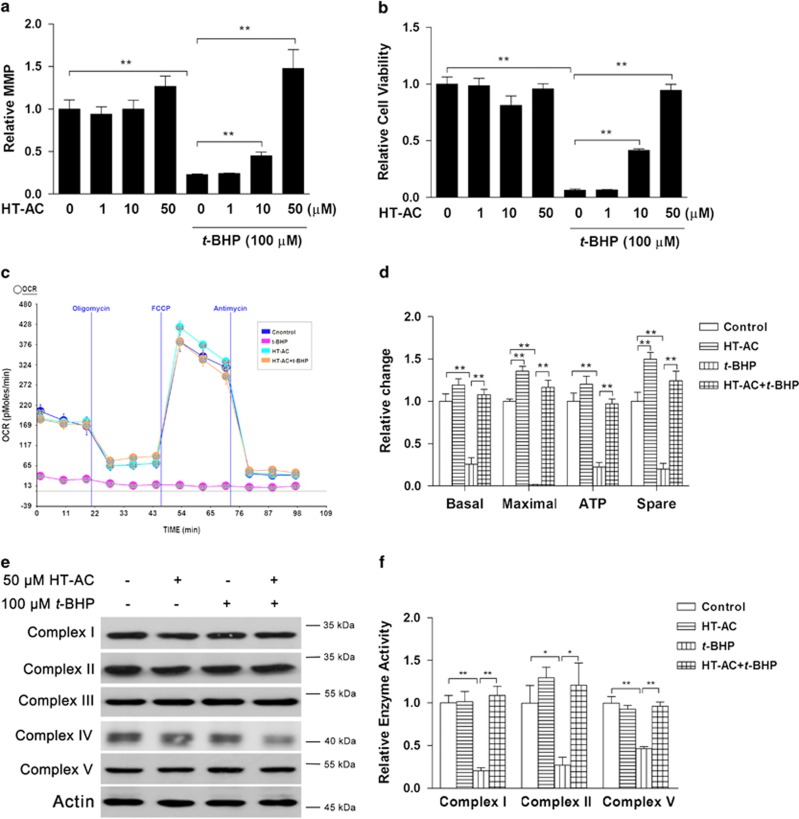
Effects of HT-AC on *t*-BHP-induced mitochondrial dysfunction. C2C12 myotubes were treated with HT-AC at doses of 1, 10 and 50 *μ*M for 24 h, followed by 100 *μ*M *t*-BHP for an additional 24 h. The protective effects of HT-AC on mitochondrial membrane potential (**a**) and cell viability (**b**) were analyzed. C2C12 myotubes were treated with 50 *μ*M HT-AC for 24 h followed by 100 *μ*M *t*-BHP for an additional 24 h. Basal, maximal and spare mitochondrial respiration capacity were analyzed as well as the ATP potential ((**c**) respiration curve; (**d**) statistical analysis). The mitochondrial complex subunits were analyzed (**e**), and complex I, II and V activities were evaluated with purified mitochondria (**f**). Values are means±S.E.M. from at least three independent experiments. *P<0.05, **P<0.01

In our previous study, we found that HT showed protective effects on strenuous exercise-induced muscle damage.^6^ Here we further investigated the potential protective effect and underlying mechanism of HT-AC in an oxidative stress-induced C2C12 cell toxicity model. As expected, pretreatment with HT-AC for 24 h showed significant protection at doses of both 10 and 50 *μ*M (Figures 6a and b), indicating that HT-AC might also be an effective nutrient. Further investigation on mitochondrial oxygen consumption and electron transport chain complex activities was conducted with HT-AC at the dose of 50 *μ*M. As shown in Figure 6c, *t*-BHP abolished mitochondrial respiration capacity including basal, maximal, ATP potential and spare respiration, all of which were significantly improved by HT-AC pretreatment (Figures 6c and d). Although mitochondrial content, evidenced by mitochondrial complex subunit expression, was not affected by either *t*-BHP or HT-AC (Figure 6e), the activities of mitochondrial complex I, II and V were decreased by *t*-BHP and then restored to normal levels by HT-AC pretreatment (Figure 6f).

### HT-AC prevents oxidative stress-induced OPA1 cleavage

**Figure 7:**
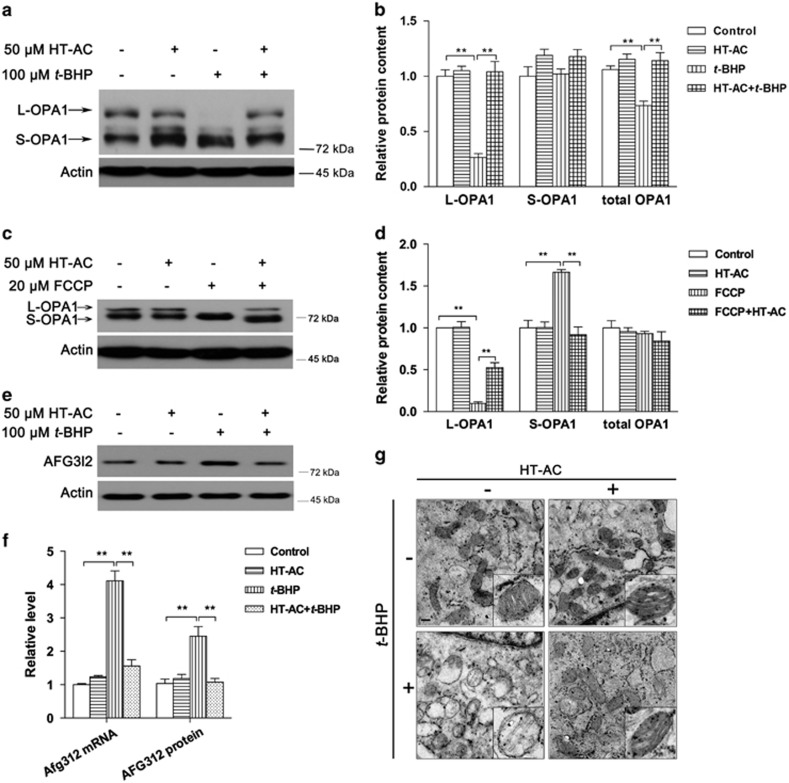
HT-AC inhibits oxidative stress-induced OPA1 cleavage. C2C12 myotubes were treated with 50 *μ*M HT-AC for 24 h followed by 100 *μ*M *t*-BHP for additional 24 h or 20 *μ*M FCCP for additional 2 h. OPA1 protein levels were analyzed after *t*-BHP treatment ((**a**) western blot image; (**b**) statistical analysis) and FCCP treatment ((**c**) western blot image; (**d**) statistical analysis). Afg3l2 mRNA and protein levels were analyzed after 6 h *t*-BHP treatment ((**e**) western blot image; (**f**) statistical analysis). Mitochondrial morphology was analyzed by transmission electron microscopy at the original magnification of × 10k or × 50k (**g**). Values are means±S.E.M. from at least three separate experiments. *P<0.05, **P<0.01

As significant mitochondrial function loss was induced by *t*-BHP, whereas mitochondrial content was not affected, we thereby assumed that mitochondrial morphology change might be the major contributor of mitochondrial dysfunction. Further analysis showed that HT-AC pretreatment could sufficiently inhibit *t*-BHP-induced OPA1 cleavage (Figures 7a and b). Although HT-AC was unable to prevent FCCP-induced MMP loss (Supplementary Figure S3), FCCP-induced ROS overproduction (Supplementary Figure S3) as well as OPA1 cleavage (Figures 7c and d) were sufficiently inhibited by HT-AC. Meanwhile, *t*-BHP-induced Afg312 increase on mRNA and protein levels was prevented by HT-AC (Figures 7e and f). As shown in Figure 7g, *t*-BHP induced significant mitochondrial structural and morphological changes, as evidenced by irregular cristae and swollen mitochondria, that were also efficiently inhibited by HT-AC pretreatment (Figure 7g).

### HT-AC inhibits oxidative stress-induced MyHC abnormality

**Figure 8:**
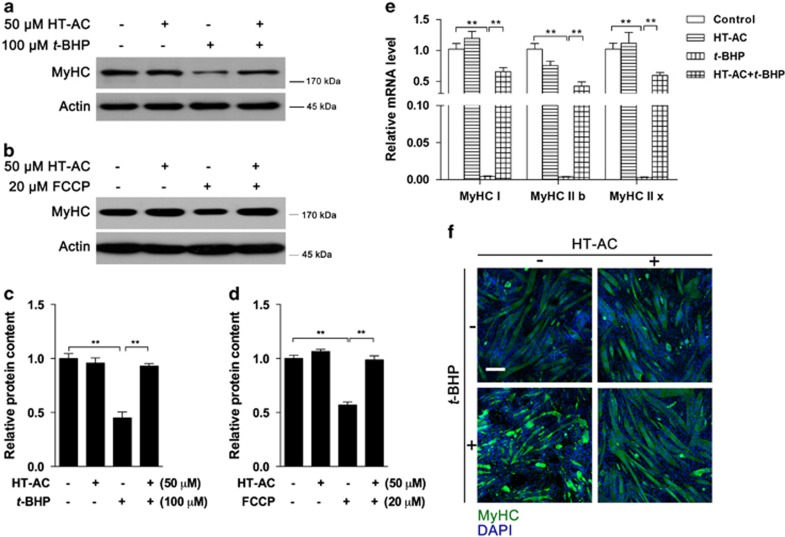
Effects of HT-AC on oxidative stress-induced MyHC abnormalities. C2C12 myotubes were treated with 50 *μ*M HT-AC for 24 h followed by 100 *μ*M *t*-BHP for additional 24 h or 20 *μ*M FCCP for additional 2 h. MyHC protein levels were determined after *t*-BHP treatment ((**a**) western blot image; (**c**) statistical analysis) and FCCP treatment ((**b**) western blot image; (**d**) statistical analysis). The mRNA levels of MyHC isoforms were analyzed by real-time PCR (**e**). MyHC distribution was observed by immunocytochemistry analysis (**f**). Values are means±S.E.M. from at least three separate experiments. *P<0.05, **P<0.01

As both OPA1 cleavage and mitochondrial dysfunction were sufficiently inhibited by HT-AC pretreatment, we thereby investigated the changes of MyHC content. As expected, the decrease in MyHC protein induced by *t*-BHP (Figures 8a and c) and FCCP (Figures 8b and d) was both significantly inhibited by HT-AC. A similar pattern was observed for the mRNA levels of the MyHC isoforms MyHC I, MyHC IIB and MyHC IIx (Figure 8e). In addition, immunocytochemistry analysis indicated that irregular distribution of MyHC, in addition to decreased protein content, and HT-AC pretreatment successfully maintained a normal distribution of MyHC in the C2C12 myotubes (Figure 8e).

## Discussion

Muscle abnormalities can lead to devastating consequences, including insulin sensitivity decline, inflammatory reactions and a decrease in exercise capacity. Among the various physiological and pathological contributions, mitochondrial dysfunction is no doubt an important one because of its role in ATP and ROS production. Recent studies have indicated that impaired mitochondrial dynamics could affect muscle cell glucose uptake and contribute to insulin resistance,^29^ and mitochondrial dynamic remodeling was closely associated with strenuous exercise-induced muscle damage. As a known regulator of mitochondrial fusion, OPA1 could affect mitochondrial morphology and cellular function through modulation of its cleavage.^15, 30, 31^ Therefore, in the current study, we investigated the involvement of OPA1 cleavage in oxidative stress-induced mitochondrial dysfunction and muscle degeneration.

*t*-BHP was employed as a model of exogenous oxidative stress for its better stability than hydrogen peroxide. Despite a structural difference, both hydrogen peroxide and *t*-BHP have been widely used in muscle and other studies.^6, 32, 33, 34^ C2C12 myotube differentiation was confirmed by time-dependent MyHC expression. After *t*-BHP treatment, rapid MMP, cell viability and ATP loss were observed, consistent with previous studies in pancreatic *β*-cells,^35^ human aortic smooth muscle cells (HASMCs) and human umbilical vein endothelial cells (HUVECs).^36^ It is interesting to note that *t*-BHP induced rapid ROS overproduction, but at 2 h, instead of accumulating, the ROS level was generally decreased. We thereby assumed that long-term treatment with t-BHP impaired mitochondrial basal respiration and led to less ROS production, and this is supported by the data in Figure 6. Along with mitochondrial dysfunction, MyHC, as well as its isoforms MyHC I, MyHC IIb and MyHC Iix, were found to be decreased significantly and accompanied by obvious myotube morphology changes. In muscle cells, myosin II generates sufficient force to complete the physiological activities through energy release from ATP hydrolysis.^37^ MyHC functions as a subunit of myosin II, and its isoform expression levels were used as markers identifying muscle fiber type.^38^ Therefore, it is obvious that oxidative stress induced significant muscle cell degeneration in this study. Because *t*-BHP was an exogenous stress, we wondered whether endogenous stress would show similar effects. FCCP was then used to inhibit mitochondrial function and induce ROS overproduction in 15 min. More efficiently, FCCP induced significant MyHC decrease in 2 h that was sufficiently inhibited by the free radical scavenger NAC. In addition, Li *et al.*^39^ reported that TNF-*α* could induce skeletal muscle protein loss through modulation of ROS content. Collectively, our data suggested that ROS overproduction-associated oxidative stress was the major effector that induced MyHC decline and potential muscle degeneration. In addition to total MyHC protein loss, the immunocytochemistry data in Figure 8 show that irregular distribution of MyHC in myotubes and the underlying mechanism warrants further investigation.

OPA1 is known to be located in the mitochondrial inner membrane and to regulate mitochondrial fusion. Mutation of OPA1 led to neuronal degeneration with mitochondrial respiration abnormalities, swollen and vacuolated mitochondrial shape and loss of cristae organization in muscle fibers.^40^ in addition, patients with OPA1 mutation showed defective mitochondrial ATP production in skeletal muscle.^41^ In the muscle fibers of aging subjects, OPA1 levels were significantly decreased, whereas other mitochondrial dynamic regulators were not affected.^42^ In this study, we found that OPA1 underwent rapid cleavage in response to both *t*-BHP and FCCP challenge, and accumulated S-OPA1 was observed at 2 h. It was indicated that abnormal OPA1 cleavage contributed to cristae disorganization,^43, 44^ and similar mitochondrial morphology was also observed after *t*-BHP treatment in this study. Regarding OPA1 cleavage, several proteases have been claimed to be responsible, and in this study, only Afg3l2 was found to be sensitive to oxidative stress and increased significantly after both *t*-BHP and FCCP treatment. Consistent with a NAC effect on MyHC content, the cleavage of OPA1 was also inhibited by NAC. Together with the data showing an OPA1 knockdown-induced MyHC decline, we speculated that ROS might affect MyHC content through the modulation of OPA1. Thus, both NAC and OPA1 siRNAs were applied in C2C12 myotubes, and the FCCP-induced MyHC decline was not inhibited by NAC under OPA1 knockdown. Our study provides evidence suggesting a close association between OPA1 cleavage and MyHC content. However, the detailed regulatory mechanisms were not explored, and this is indeed a limitation of this study. Further investigations should focus on understating how abnormal OPA1 cleavage contributes to MyHC decline.

Despite a limited understanding of mechanisms accounting for muscle disorders, effective nutritional intervention seems to be a better way to maintain health. As a major polyphenolic compound in olive oil, HT was found effective in metabolic syndrome,^10^ unloading-induced muscle atrophy^45^ and strenuous exercise-induced muscle damage.^6^ In this study, HT-AC, another effective compound that has recently been identified, was investigated. As expected, HT-AC significantly prevented *t*-BHP-induced OPA1 cleavage as well as the induction of Afg3l2. The significant morphology change in mitochondria induced by *t*-BHP was also normalized by HT-AC. Analysis of mitochondrial function revealed significant protection by HT-AC on mitochondrial basal, maximal, spare respiration and complex activities. Because the mitochondrial complex subunits were not affected by either *t*-BHP or HT-AC, we concluded that the decreased mitochondrial function was potentially attributable to morphology changes. More importantly, the decrease in MyHC protein content and mRNA content, as well as the irregular distribution of MyHC in C2C12 myotubes, were all sufficiently normalized by HT-AC, suggesting that HT-AC may be an efficient nutrient preventing mitochondrial dysfunction-associated muscle disorders.

Taken together, the results of the current study indicate that oxidative stress induced significant mitochondrial dysfunction and MyHC protein loss, potentially by modulating OPA1 cleavage. In addition, HT-AC works as an effective nutrient preventing OPA1 cleavage, mitochondrial dysfunction and muscle cell degeneration. Hopefully, potential new targets and effective nutrients provided by further study could contribute to the development of new strategy for the prevention and treatment of muscle disorders.

## Materials and Methods

### Chemicals

*t*-BHP, FCCP, antimycin A, oligomycin and ATP assay kit were from Sigma (St. Louis, MO, USA). Fetal bovine serum (FBS) was from PAA Laboratories GmbH (Linz, Austria). High glucose Dulbecco's modified Eagle's medium (DMEM), horse serum (HS), JC-1 (5,5,6,6-tetrachloro-1,1,3,3-tetraethylbenzimidazolylcarbocyanine iodide), DAPI and antibodies against complex I, II, III, IV and V were from Invitrogen (Carlsbad, CA, USA). HRP-conjugated anti-mouse/rabbit IgG antibodies were purchased from Jackson ImmunoResearch Laboratories (West Grove, PA, USA). The antibodies against MyHC, AFG312, PARP and cleaved PARP were from Santa Cruz Biotechnology (Santa Cruz, CA, USA). The antibodies against *β*-actin, cleaved caspase 3 and cleaved caspase 9 were from Cell Signaling Technology (Danvers, MA, USA). The antibody against OPA1 was from BD (Franklin Lakes, NJ, USA). OPA1 siRNA oligo and PCR primers for Afg3l2, MyHC I, MyHC IIb, MyHC IIx, Murf-1, Atrogin-1 and *β*-actin were synthesized by Genepharma (Shanghai, China).

### Cell culture

Mouse C2C12 myoblasts were purchased from the ATCC (Manassas, VA, USA) and cultured in DMEM supplemented with 100 U/ml penicillin and 100 *μ*g/ml streptomycin and 10% FBS. Cell cultures were maintained at 37°C in a humidified atmosphere of 95% air and 5% CO_2_. Medium was changed every 2 days. For the study of muscle cells, cells were differentiated as previous report.^6^

### MTT assay for cell viability

C2C12 myoblasts were cultured in 96-well plates. After differentiation and treatment, cell viability was determined by the addition of MTT (3-[4,5-dimethylthiazol-2-yl]-2,5-diphenyltetrazolium bromide). Optical densities were read at 555 nm using a microplate spectrophotometer (Multiskan Ascent, Thermo Fisher Scientific Inc., Waltham, MA, USA).

### JC-1 assay for MMP

C2C12 myoblast were cultured in 96-well plates. After differentiation and treatment, MMP was detected with JC-1. For quantitative fluorescence measurement, cells were incubated with JC-1 staining and scanned using a microplate fluorometer (Fluoroskan Ascent, Thermo Fisher Scientific Inc.) at 488 nm excitation and 535 and 590 nm emission wavelengths to measure green and red JC-1 fluorescence, respectively. The red/green fluorescence intensity ratio reflects MMP.

### Intracellular ATP levels

C2C12 myoblasts were cultured in six-well plates. After differentiation and treatment, cells were lysed with 0.5% Triton X-100 in 100 mM glycine buffer, pH 7.4. Intracellular ATP level assays were performed using an ATP assay kit following the manufacturer's protocol. ATP was consumed and light was emitted when firefly luciferase catalyzed the oxidation of D-luciferin.^46^

### ROS measurement

C2C12 myoblasts were cultured in six-well plates. After differentiation and treatment, the generation of intracellular ROS was determined using the fluorescence of 2̀,7̀-dichlorofluorescein (H2DCF-DA). H2DCF-DA was incubated with live cells in serum-free medium for 45 min, and then cells were collected with PBS. After centrifugation at 1000 *g* for 5 min at 4ºC, cells were suspended with cold PBS. Cells were analyzed by flow cytometry (BD Biosciences, Franklin Lakes, NJ, USA).

### Cell oxygen consumption rate (OCR) measurement

C2C12 myoblasts were seeded in XF 24-well microplates (Seahorse Bioscience, Billerica, MA, USA). After differentiation and treatment, oxygen consumption was measured with extracellular flux analysis (Seahorse Biosciences). The final concentrations of mitochondrial inhibitors were at 10 *μ*M antimycin A, 6 *μ*M FCCP and 10 *μ*M oligomycin. Basal respiration is the baseline oxygen consumption reading before compounds are injected. Maximal respiration represents the maximum OCR measurement value after FCCP injection. Spare respiratory capacity is calculated by noting the OCR response to FCCP, and dividing that number by the basal respiration to get a percentage. After detection, cells numbers were calculated and OCR was adjusted accordingly.

### Transmission electron microscope (TEM) assays

C2C12 myoblasts were cultured in six-well plates. After differentiation and treatment, cells were washed with PBS and then collected for centrifugation at 1000 *g* for 10 min at 4ºC. The supernatant was discarded, and cells were fixed with 2.5% glutaraldehyde in 0.1 M phosphate buffer and the TEM assay was performed following a previous study.^45^

### Myotube mitochondrial isolation

C2C12 myoblast were seeded in 10-cm dishes. After differentiation and treatment, cells were washed with cold PBS and collected for centrifugation at 1000 *g* for 10 min at 4ºC. The supernatant was discarded and the pellet was resuspended in a hypotonic RSB buffer (10 mM NaCl, 2.5 mM MgCl_2_, 10 mM Tris base, pH 7.5) and allowed to swell. The swollen cells were homogenized with a Dounce homogenizer. MS buffer (210 mM mannitol, 70 mM sucrose, 5 mM Tris base, 1 mM EDTA, pH 7.5) was added and then the mixture was centrifuged at 1000 *g* for 10 min at 4ºC. The pellet was then centrifuged at 17 000 *g* for 15 min at 4°C to obtain the mitochondrial pellet. The pellet was resuspended with isolation buffer (Tris base 100 mM, sucrose 100 mM, EDTA 10 mM, KCl 46 mM, BSA, 0.5% (W/W), pH 7.5) and stored at −80°C for further analysis.

### Mitochondrial complex activity assays

NADH-ubiquinone reductase (complex I), succinate-CoQ oxido-reductase (complex II), CoQ-cytochrome *c* reductase (complex III), cytochrome *c* oxidase (complex IV) and ATP synthase (complex V) activities were measured spectrophotometrically using conventional assays following a previous report.^46, 47^ All of the activities were adjusted by the expression level of each complex.

### MyHC immunocytochemistry analysis

C2C12 myoblasts were cultured on cover glass discs in six-well plates. After differentiation and treatment, cells were washed with PBS and then fixed with 4% paraformaldehyde in PBS for 20 min at room temperature. After being rinsed with PBS, the cells were permeabilized with 0.25% Triton X-100 for 10 min at room temperature and then blocked with 1% BSA in PBST for 1 h at room temperature and washed with PBS. Cells were then incubated with antibody against MyHC (1 : 500) in 1% BSA overnight at 4°C and further incubated with FITC-labeled Goat Anti-Rabbit IgG (Beyotime, Jiangsu, China) in 1% BSA for 1 h at room temperature in the dark. After washing with PBS, the cells were incubated with 0.5 *μ*g/ml DAPI for 5 min and visualized by confocal microscopy (Zeiss, Jena, Germany).

### Western blot analysis

Samples were lysed with western and IP lysis buffer (Beyotime). The lysates were homogenized and centrifuged at 13 000 *g* for 10 min at 4°C. The supernatants were collected and the protein concentrations were determined using the BCA Protein Assay kit. Equal amounts (10 *μ*g) of each protein sample were applied to SDS-PAGE gels, transferred to pure nitrocellulose membranes (PerkinElmer Life Sciences, Boston, MA, USA) and blocked with 5% nonfat milk. The membranes were incubated with the first antibody at 4°C overnight. Then, the membranes were incubated with anti-rabbit or anti-mouse secondary antibodies at room temperature for 1 h. Chemiluminescent detection was performed using an ECL western blotting detection kit (Thermo Fisher, Rockford, IL, USA). The results were analyzed by Quantity One software (Bio-Rad, Shanghai, China) to obtain the optical density ratio of target proteins relative to *β*-actin.

### SiRNA transfection

Transfection with siRNA targeting OPA1 was performed using the target sequence from mouse OPA1, and scrambled siRNA was used as negative control. After differentiation in six-well plates, myotubes were prepared for transfection. The transfection was performed using Lipofectamine 2000 (Invitrogen), as described in the supplier's manual. SiRNA (200 pmol) was incubated per well containing serum-free DMEM for 24 h. The medium was replaced with DMEM containing 2% HS for another 48 h, and then the cells were treated with different conditions.

### Real-time PCR

Total RNA was extracted from the cells using TRIzol reagent (Roche, Basel, Switzerland) following the manufacturer's protocol. Reverse transcription was performed using the PrimeScript RT-PCR Kit (Otsu, Shiga, Japan) followed by semi-quantitative real-time PCR using specific primers. The primer sequences were as follows: Afg3l2, 5′-AAAACTCCGGTTGATGGGCA-3′ (forward) and 5′-CCGGTTCTCCCCTTCTATGC-3′ (reverse); MyHC I, 5′-CCAGGGGCAAACAGGCATTCACT-3′ (forward) and 5′-CTTCCACTGGGCCACTTCACTGTT-3′ (reverse); Mrf4, 5′-GCAGAGGGCTCTCCTTTGTA-3′ (forward) and 5′-GGTAGCTGCTTTCCGACGAT-3′ (reverse); Myod, 5′-CCAGGACACGACTGCTTTCT-3′ (forward) and 5′-TCTGGTGAGTCGAAACACGG-3′ (reverse); Myogenin, 5′-GAGACATGAGTGCCCTGACC-3′ (forward) and 5′-AGGCTTTGGAACCGGATAGC-3′ (reverse); MyHC IIb, 5′-GTGATTTCTCCTGTCACCTCTC-3′ (forward) and 5′-GGAGGACCGCAAGAACGTGCTGA-3′ (reverse); MyHC IIx, 5′-TGAAGGGCGGCAAGAAGCAGAT-3′ (forward) and 5′-GCGGAATTTGGCCAGGTTGACA-3′ (reverse); *β*-actin 5′-ACGGCCAGGTCATCACTATTG-3′ (forward) and 5′-CACAGGATTCCATACCCAAGAAG-3′ (reverse).

### Statistical analysis

The results are presented as the mean±S.E.M. Statistical analyses were conducted using one-way ANOVA followed by least significant difference *post hoc* analysis or an unpaired *t-*test. For all analyses, values of *P*<0.05 were considered statistically significant.

## Abbreviations

Afg312: ATPase family gene 3-like 2
FCCP: carbonyl cyanide 4-(trifluoromethoxy) phenylhydrazone
HT-AC: hydroxytyrosol acetate
MyHC: myosin heavy chain
Myod: myogenic differentiation 1
NAC: *N*-acetyl-L-cysteine
OPA1: optic atrophy 1
*t*-BHP: *tert*-butylhydroperoxide
