# Supplementary material for: Mitochondrial dysfunction-associated OPA1 cleavage contributes to muscle degeneration: preventative effect of hydroxytyrosol acetate
Source: Cell Death Dis. 2014 Nov 13;5(11):e1521–. doi: 10.1038/cddis.2014.473 (PMC4260731; doi:10.1038/cddis.2014.473)
Supplement: Supplementary Information [file cddis2014473x1.doc]

**Supporting Information**

1. **Effects of t-BHP on C2C12 myoblast cell viability and mitochondrial membrane potential (MMP)**

**
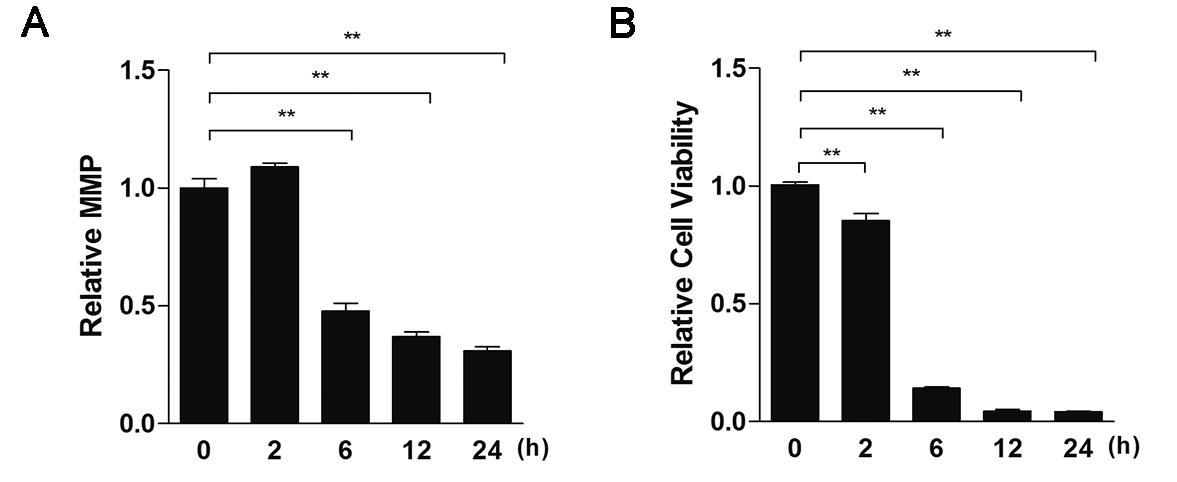
**

**Fig S2. Effects of t-BHP on C2C12 myoblast.** C2C12 myoblast were treated with 100 μM *t*-BHP for the indicated time periods, and mitochondrial membrane potential (A), and cell viability (B) were detected. The values are means ± S.E.M. from at least three independent experiments. *p< 0.05, **p< 0.01.

1. **Effects of *t*-BHP on Murf-1 and Atrogen-1 mRNA expression in C2C12 myotubes**

**
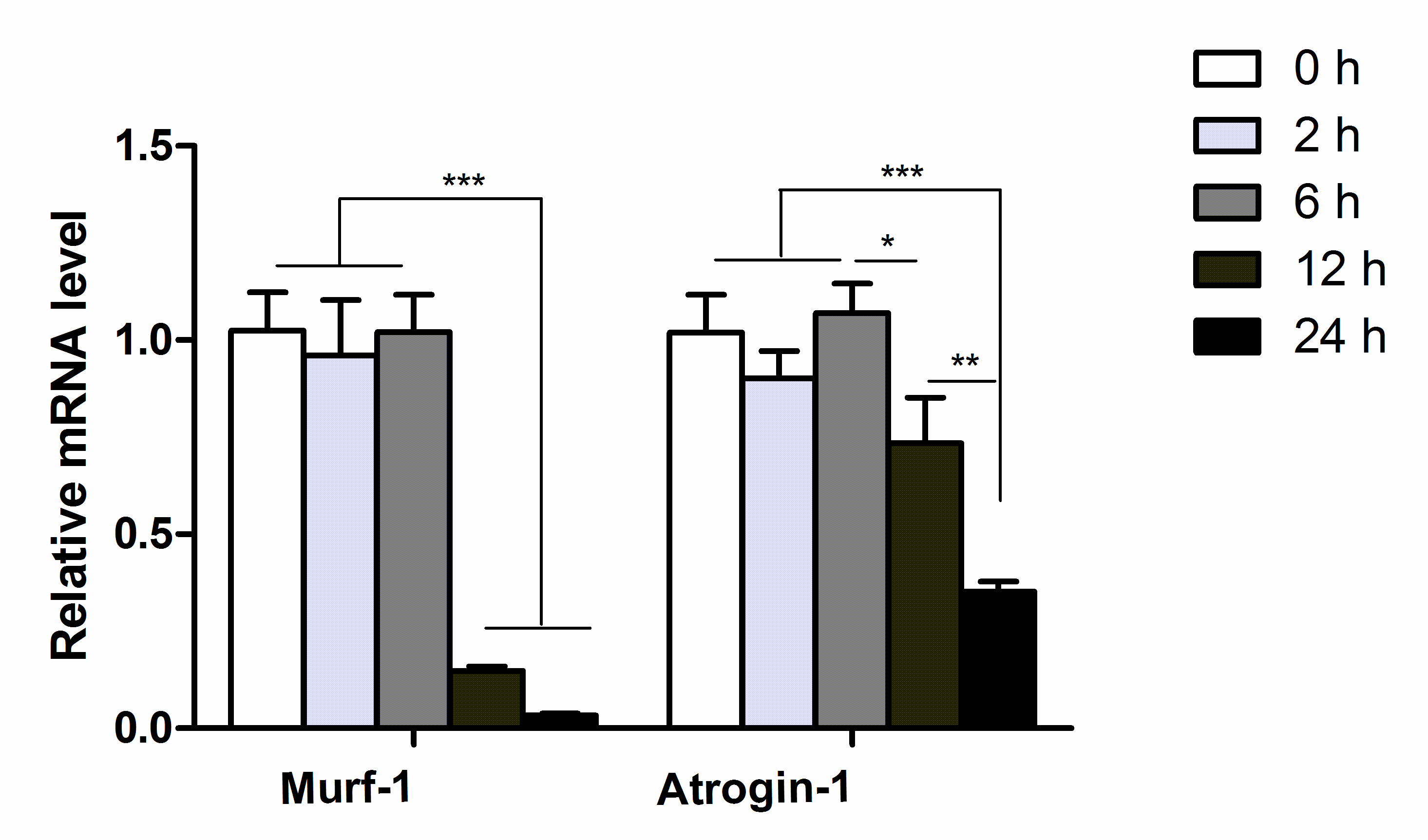
**

**Fig S1. Effects of *t*-BHP on Murf-1 and Atrogen-1 mRNA levels.** C2C12 myotubes were treated with 100 μM *t*-BHP for the indicated time periods, mRNA expression of Murf-1 and Atrogin-1 was analyzed by real-time PCR. The values are means ± S.E.M. from at least three independent experiments. *p< 0.05, **p< 0.01.

1. **Effects of HT-AC and FCCP on C2C12 myotubes**

**
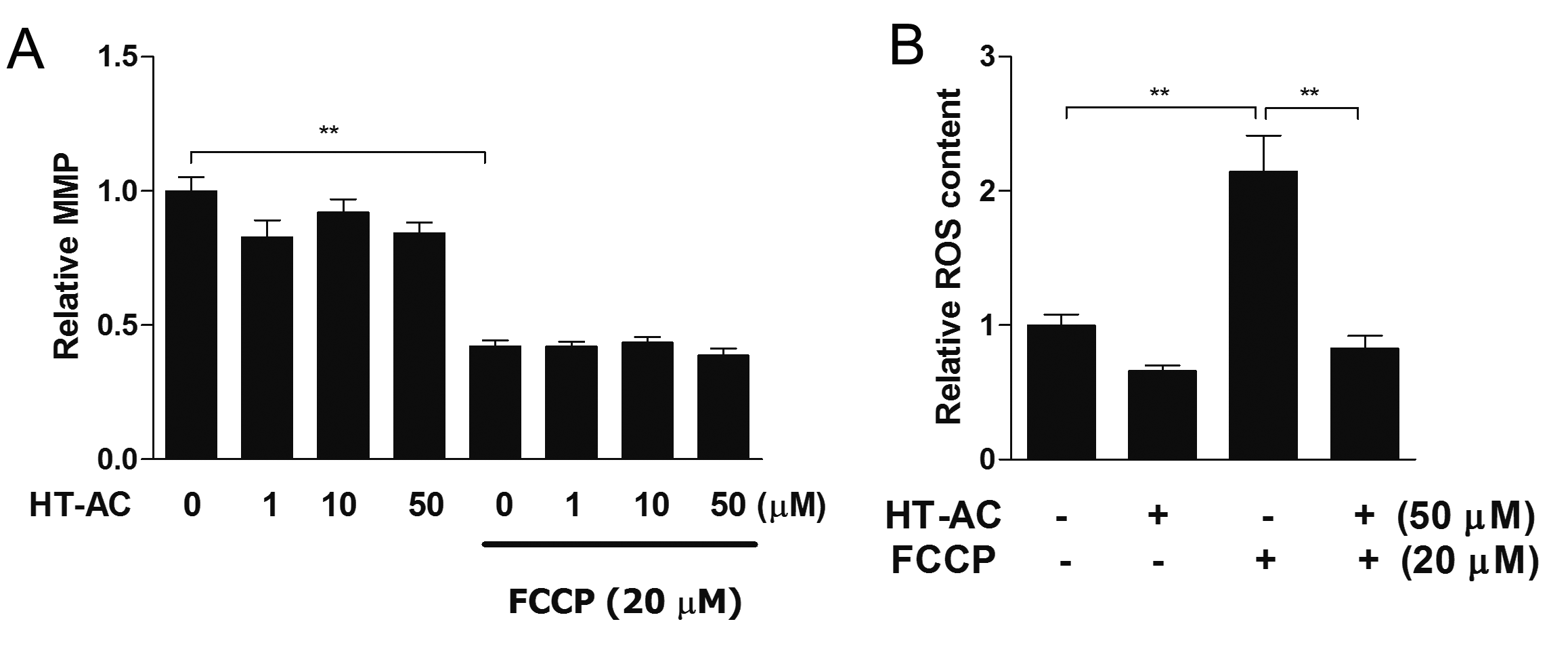
**

**Fig S3. Effects of HT-AC and FCCP on MMP and ROS.** (A) C2C12 myotubes were pretreated with 1, 10, 50 μM for 24 h, followed by 20 μM FCCP treatment for another 24 h, MMP was then measured. (B) C2C12 myotubes were pretreated with 50 μM HT-AC for 24 h, followed by FCCP treatment for 15 min, ROS content was then measured. The values are means ± S.E.M. from at least three independent experiments. **p< 0.01.
